# The Pay of Panel Doctors

**Published:** 1920-01-24

**Authors:** 


### THE PAY OF PANEL DOCTORS.

Labour organisations have always been very ready to justify their policy of collective bargaining and insistence upon their demands by pointing to the professional organisations which protect the interests of lawyers, doctors, and others. It might be well if they also followed examples set by the professional organisations, such as that just provided by the doctors. The Conference of Local Medical and Panel Committees had resolved on an increased yearly capitation fee of 13s. 6d. and the Government offered 11s., but the doctors asked for arbitration, to which the Government consented, with the promise that Parliament would be recommended to give effect to the findings of the arbitrators. The Minister of Health reserved his freedom to institute, at a later date, any inquiry which might be thought desirable, in the light of the award and the cost of its working, into the question whether as good or better a service could be secured with the same or less expenditure of money under some other scheme. Meanwhile, the Government agrees to pay a capitation fee of 11s. from last January 1, and the arbitrators' award will operate as from next April.

### Higher Benefits and Contributions.

The Ministry of Health announces that a Bill will be introduced into Parliament at the first opportunity for increased benefits and contributions under the National Health Insurance scheme.

The weekly contributions are to be increased by 3d. in the case of both men and women, 2d. of which is to fall on the employer and 1d. on the employed person.

The normal rate of sickness benefit is to be increased to 15s. for men and 12s. for women. Disablement benefit is to be at the rate of 7s. 6d. a week for both men and women, and maternity benefit is to be increased from 30s. to £2. Sanatorium benefit is to be removed from the Acts, the treatment (other than domiciliary) of tuberculosis, both among the insured and the uninsured, being recognised as falling within the province of the local authorities.

A State system of medical referees is to be established, towards the cost of which the societies will make a small contribution by way of payment per case referred.

The increased contributions, together with the consequential increase in the Exchequer contributions, will, in addition to providing for the increases in the rates of cash benefits, enable a larger sum than hitherto to be made available towards meeting the increased cost of medical benefit.

It is not clear whether this increased cost of medical benefit includes the extra fees to the doctors. Presumably it does not, seeing that the arbitration has yet to be effected.

